# Aneurysm of the Pulmonary Artery in Fallot's Tetralogy

**DOI:** 10.1155/2017/1384905

**Published:** 2017-05-22

**Authors:** Kawtar Afrikh, Loua Hattach, Nadia Fellat, Mustapha El Bakkali, Halima Benjelloun

**Affiliations:** ^1^Unit of Cardiology A, Ibn Sina University Hospital, Rabat, Morocco; ^2^Physiology of Exercise Team (EPE), Faculty of Medicine and Pharmacy, University Mohammed V, Rabat, Morocco

## Abstract

**Introduction:**

Pulmonary artery aneurysms are a rare entity. Etiologies of these findings are multiple, but they are exceptionally associated with Fallot's Tetralogy. In this study, we present an unusual case of an important aneurysm of the left pulmonary artery associated with Fallot's Tetralogy disease.

**Case Presentation:**

A 30-year-old woman has been admitted for dyspnea and cyanosis. The data which had been obtained from echocardiography, cardiac catheterization, and angio-magnetic resonance imaging (MRI) suggested the existence of an important aneurysm of the left pulmonary artery associated with a regular Fallot's disease with a pulmonic stenosis. We have noticed the presence of a small restrictive patent ductus arteriosus (PDA). Therefore, the patient was referred to surgical correction.

**Conclusion:**

Pulmonary artery aneurysms associated with Fallot's Tetralogy are rarely reported. The natural history of these rare arterial aneurysms has to be clarified.

## 1. Introduction

Aneurysms of the pulmonary artery (PAA) are rarely reported in the literature [1]. Predisposing conditions include congenital and acquired heart disease, such as left-to-right shunting or pulmonary valve stenosis with poststenotic dilatation. Other causes include infections (tuberculosis, syphilis, osteomyelitis, and pneumonia), systemic vascularities (Hughes-Stovin's disease, Behcet's disease), collagen vascular diseases, connective tissue disorders, inherited disorders (Marfan's syndrome, Ehlers-Danlos syndrome), trauma (direct or blunt chest injury), mucoid vasculopathic changes, and idiopathic PAA. The diagnosis of these entities has been improved by the imaging methods such as echocardiography, computed tomography (CT), and magnetic resonance imaging (MRI). Natural history of PAAs remains largely unknown and, up to now, guideline recommendations are not established for the optimal treatment.

## 2. Case Presentation

**Figure 1:**
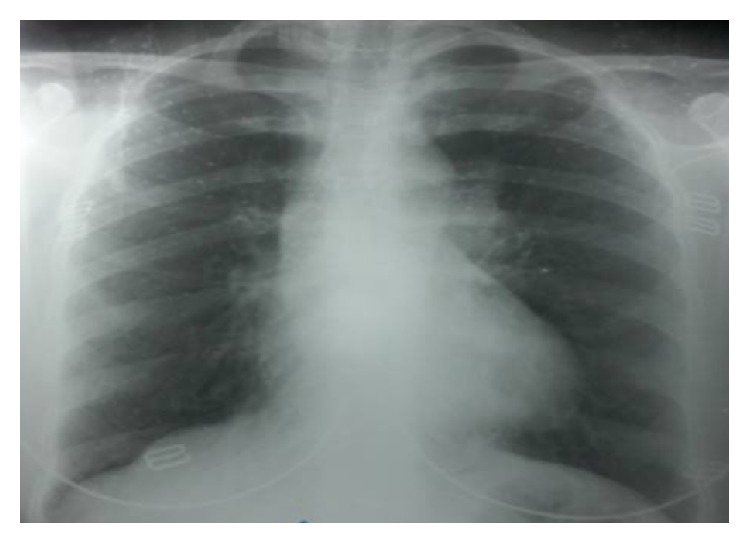
Posteroanterior chest X-ray showing a mild increase in cardiothoracic ratio.

**Figure 2:**
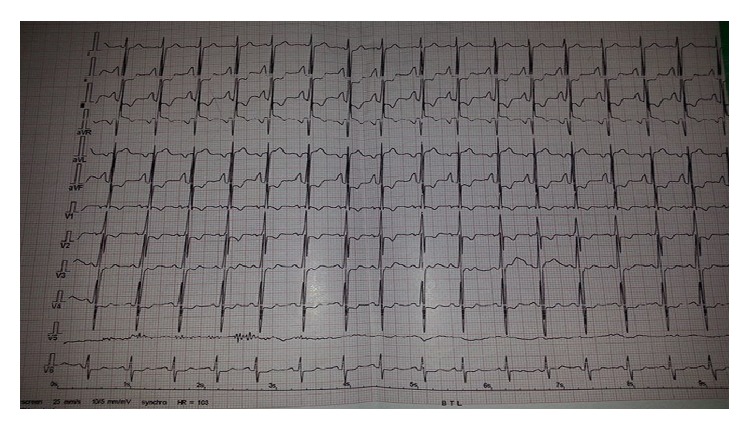
Electrocardiogram showing right ventricular hypertrophy and a right auricular hypertrophy.

We report a 30-year-old female patient recently diagnosed with PAA associated with Fallot's Tetralogy disease. She has been admitted to our clinic with the complaints of dyspnea and cyanosis. She has no infection medical history or trauma. A written consent form was obtained from the patient before the action; in addition the present study was approved by the Ibn Sina Ethical Committee after a thorough analysis. Clinical examination has revealed a 3/6 systolic ejection murmur on the left sternal border. There is no evidence of heart failure. Chest X-ray has shown a mild increased cardiothoracic ratio with coeur-en-sabot appearance associated with an unusual enlargement of the middle left cardiac border (Figure 1). Also, the electrocardiogram has exposed a right ventricular hypertrophy and a right auricular hypertrophy (Figure 2).

Transthoracic echocardiography examination has revealed a congenital heart disease with characteristics of Fallot's Tetralogy including perimembranous Ventricular Septal Defect, an overriding aorta, left and right ventricular hypertrophy, and pulmonary valvular stenosis (mean gradient = 64 mmHg), without pulmonary regurgitation. In addition, dilatation of the left pulmonary artery has also been detected suggesting a poststenotic impact on the pulmonary artery. Cardiac catheterization has been performed. The hemodynamic results have made known the classic similar systolic pressure of the ventricular chambers and the aorta. The pulmonary arterial systolic pressure was 27 mmHg and the mean pulmonary arterial pressure was 17 mmHg (normal < 25 mmHg) [2].

**Figure 3:**
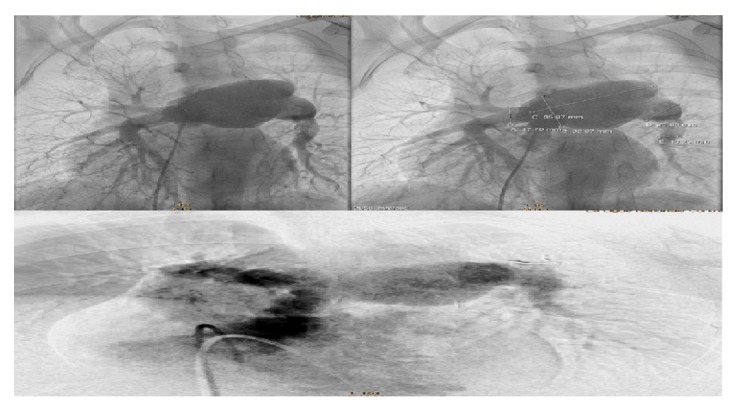
A pulmonary artery angiogram revealed a large aneurysm of the left pulmonary artery (47/65 mm) associated with a valvular stenosis.

**Figure 4:**
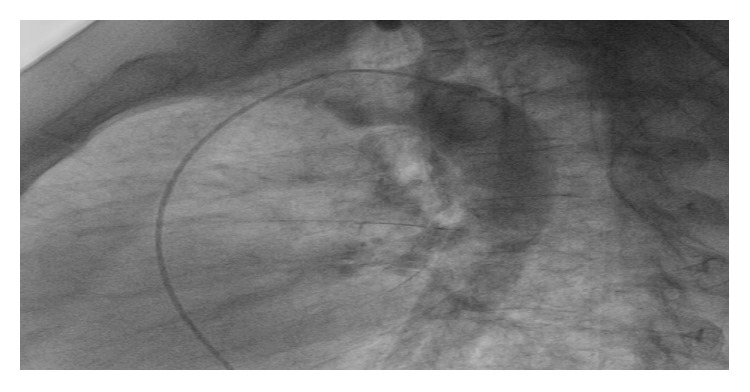
Aortic angiogram showing presence of a small PDA.

Pulmonary arterial angiography indicated a disharmonious pulmonary tree with a marked dilation of left pulmonary artery (dimensions = 47/65 mm) (Figure 3). There was a valvular obstacle with no pulmonary regurgitation. We have noticed the presence of a small restrictive PDA (Figure 4).

**Figure 5:**
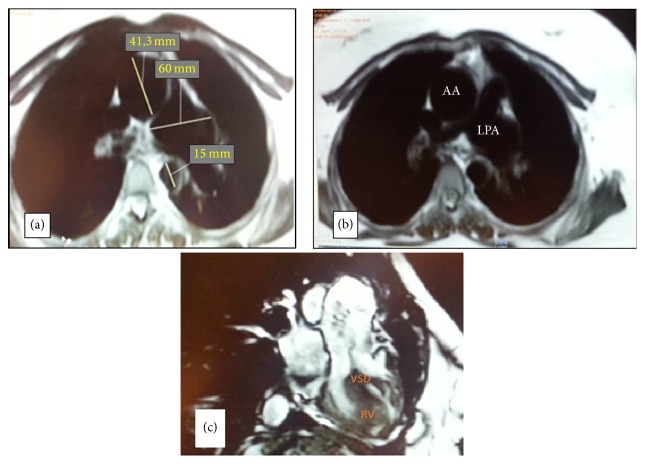
(a and b) The transverse section of a chest MRI showed an aneurysmal change in the left pulmonary artery. Dimensions of the main and right pulmonary arteries were normal. The ascending aorta was also mildly dilated. (c) Cine cardiac magnetic resonance image showing lesions of Fallot's Tetralogy (VSD, overriding aorta, and dilated right ventricle).

An angio-MRI was also performed. It showed a Fallot's Tetralogy with a dilated right ventricle associated with an aneurysm of the left pulmonary artery (Figures 5(a), 5(b), and 5(c)). Patient was referred to surgical correction.

## 3. Discussion

Pulmonary artery aneurysm is a rare disease. About half of the cases are associated with congenital heart diseases, such as patent ductus arteriosus, atrial septal defects, and ventricular septal defects that cause volume and pressure overloading in the right cardiac cavities [3]. This pathology can also occur due to inflammatory changes in the artery walls resulting from mycosis and syphilis, Behcet's disease, and trauma [4, 5]. Isolated aneurysms may be existing with no known pathologies, but they are a very rare entity.

PAAs are seen in most cases (66%) with a pulmonary arterial hypertension [1], regardless of the underlying cause. They are mostly asymptomatic. Symptoms are especially related to complications: bronchial or tracheal compression, dissection, rupture, or thrombus (caused by reduced blood flow velocity). The aneurysm can be discovered incidentally on chest radiography for unexplained dyspnea [6].

Pulmonary arterial angiography is the gold standard test in diagnosis of PAA. Other recent technical modalities can also be used for optimal imaging in the workup such as echocardiography, MRI, and spiral CT which can demonstrate the patent lumen and detect any mural thrombus or other abnormalities of the vessel wall [1, 7].

PAAs in Fallot's Tetralogy are rarely reported. Only three cases are found in the literature [8–10]. Fallot's disease usually gives a small and harmonious pulmonary tree. Echocardiographic findings and data that are obtained from cardiac catheterization and angio-MRI in the present case have suggested the existence of an important aneurysm of the left pulmonary artery associated with a regular Fallot's disease with a pulmonic stenosis. The patient has no agenesis of pulmonic valve.

A hypothesis of having a mycotic aneurysm which has developed secondary to bacterial endarteritis superimposed upon a patent ductus arteriosus is also probable. But no proof has been found. It should be noted that the association of Fallot's Tetralogy to a PDA is exceptional.

## 4. Conclusion

The etiologies of pulmonary artery aneurysms are multiple. But these entities are rarely associated with Fallot's Tetralogy, as far as the present case is concerned. Some hypotheses have been discussed, but the natural history of these rare arterial aneurysms has to be clarified.
